# Efficacy and Safety of Switching From Intravenous to Subcutaneous Vedolizumab in Japanese Patients With Ulcerative Colitis: A Study Focusing on Injection‐Site Reactions

**DOI:** 10.1002/jgh3.70407

**Published:** 2026-04-21

**Authors:** Makoto Furuya, Kiichiro Yoza, Takeshi Yonezawa, Ken Takeuchi

**Affiliations:** ^1^ Department of Gastroenterology IBD Center, Tsujinaka Hospital Kashiwanoha Chiba Japan; ^2^ Department of Gastroenterology Graduate School of Medicine, Chiba University Chiba Japan

**Keywords:** injection‐site reactions, real‐world data, subcutaneous injection, ulcerative colitis, vedolizumab

## Abstract

**Background/Aims:**

The subcutaneous (SC) formulation of vedolizumab is available as an alternative to the intravenous (IV) formulation in patients with ulcerative colitis (UC). However, data from Asian patients are limited, and the clinical course of injection‐site reactions (ISRs) remains unclear. This study evaluated the efficacy and safety of switching from IV to SC vedolizumab in Japanese patients with UC, focusing on ISRs.

**Methods:**

This retrospective study included patients who switched from IV to SC vedolizumab. The primary outcomes were the incidence and clinical course of ISRs. The secondary outcomes included adverse events other than ISRs, disease activity, remission rates, and treatment persistence.

**Results:**

In total, 48 patients were included. The median fecal calprotectin levels remained stable from baseline to 12 months after switching (46.0 vs. 46.0 μg/g), with the fecal calprotectin remission rate remaining stable (41/45, 91% vs. 39/45, 87%). The persistence rate of SC vedolizumab was 98%, and the persistence rate without additional therapy was 94%. ISRs occurred in 29 patients (60%), most frequently within 3–6 months, with a median duration of 4 months. More than half of ISRs improved or resolved by 12 months, and only 11 patients (23%) experienced persistent ISRs. No adverse events other than ISRs were considered likely or probably related to SC vedolizumab.

**Conclusions:**

In Asian patients with UC, disease activity was maintained after switching from IV to SC vedolizumab. Although ISRs were common after switching, most were self‐limiting. Therefore, switching back to IV formulation solely for ISR management should be cautiously considered.

## 1 Introduction

Ulcerative colitis (UC), along with Crohn's disease, is a chronic inflammatory bowel disease (IBD) characterized by symptoms such as abdominal pain, diarrhea, bloody stool, and fecal urgency [1]. These manifestations significantly affect patients' quality of life and interfere with social activities [2]. The prevalence of UC has also been rapidly increasing in Asian countries, including Japan [3]. Over the past decade, the development of biologic agents with diverse mechanisms of action has markedly expanded therapeutic options for UC [4]. Among these agents, vedolizumab, a gut‐selective α4β7 integrin antagonist, is widely used worldwide as an effective and safe treatment for patients with moderate to severe UC [5, 6].

Vedolizumab was initially available only as an intravenous (IV) formulation. Regular IV administration of vedolizumab can be inconvenient for some patients because of the time required for hospital visits and travel, which may negatively affect quality of life [7, 8, 9]. Repeated IV administration also requires infusion facilities, trained staff, and single‐use medical supplies, which add pressure to healthcare systems. Accordingly, IV administration has posed various challenges for both patients and healthcare providers. Based on the VISIBLE 1 trial, subcutaneous (SC) vedolizumab is an effective and safe maintenance therapy for patients with moderate to severe active UC. Hence, it has been used in real‐world clinical practice [10]. Several studies have also reported the efficacy and safety of switching from IV to SC vedolizumab in clinical settings [11, 12, 13, 14, 15].

However, most reports are from Western countries, and data from Asian patients are still limited. To date, real‐world evidence from Asia has been restricted to a single report from Korea, which focused on treatment persistence after switching from IV to SC vedolizumab [16]. Previous studies have not compared IV and SC formulations in Asian patients. Injection‐site reactions (ISRs) may occur after SC therapy and can influence clinical decisions about whether to continue SC administration or switch back to IV treatment. Nevertheless, their clinical course—including time to onset, duration, and temporal changes—has not been completely elucidated.

Therefore, the current study evaluated the efficacy and safety of switching from IV to SC vedolizumab in Japanese patients with UC in real‐world clinical practice, with a particular focus on ISRs.

## 2 Methods

### 2.1 Study Design and Patients

This retrospective observational study included patients who were on established IV vedolizumab therapy administered every 8 weeks (defined as having received ≥ 4 previous IV doses) and were subsequently switched to SC vedolizumab at Tsujinaka Hospital Kashiwanoha between June 2024 and November 2024. The clinical outcomes were observed over a 12‐month follow‐up period or until discontinuation of SC vedolizumab. Maintenance IV vedolizumab was administered at a dose of 300 mg every 8 weeks. Meanwhile, SC vedolizumab was administered at a dose of 108 mg every 2 weeks. Switching to SC vedolizumab was performed at the scheduled time of the next IV infusion (8 weeks after the last IV infusion), and no bridging therapy or treatment overlap was used.

The diagnosis of UC was made according to the consensus guidelines of the European Crohn's and Colitis Organization [17]. Clinical activity was assessed using the partial Mayo (pMayo) score, and clinical remission was defined as a pMayo score of ≤ 2 with a rectal bleeding subscore of 0 [18]. The exclusion criteria included patients who had received ≤ 3 prior IV vedolizumab doses, those with a history of colectomy involving ≥ 50% of the colorectum, or those with a confirmed diagnosis of Crohn's disease or indeterminate colitis.

### 2.2 Outcomes and Data Collection

The primary outcomes were the incidence and clinical course of ISRs (erythema, swelling, and itching), including time to onset, duration, and temporal changes (improvement or persistence) over a 12‐month observation period. The secondary outcomes, which were assessed at the 12‐month follow‐up unless otherwise specified, were as follows: (a) all adverse events during the whole observation period, including ISRs, regardless of severity, duration, or suspected association with treatment; (b) changes in disease activity, as measured by fecal calprotectin (FCP) levels (μg/g), C‐reactive protein (CRP) levels (mg/dL), and pMayo score; (c) changes in remission rates, defined as FCP levels < 150 μg/g, CRP levels < 0.3 mg/dL, and clinical remission [19]; and (d) treatment persistence of SC vedolizumab, reasons for discontinuation, and the proportion of patients who remained on SC vedolizumab without requiring any additional therapy, including systemic or budesonide‐based corticosteroids, calcineurin inhibitors, immunomodulators, or advanced therapies.

All data were collected from the medical records of the patients. Data on baseline characteristics, including sex, age, disease duration, pMayo score, disease extent, CRP and FCP levels, concomitant medications, duration of previous vedolizumab use, and history of advanced therapies, were obtained prior to switching to SC vedolizumab.

ISRs were analyzed using data obtained from routine clinical practice, including patient‐initiated telephone contacts, interviews conducted by IBD nurses during outpatient visits, and physician interviews, all of which were documented in the medical charts. Outpatient visits were generally scheduled every 2–3 months during the observation period. During each outpatient visit, patients were asked about ISR symptoms since the previous visit, including symptoms that had occurred outside the visit month whenever possible. The month of ISR onset was defined as the month in which the patient first noticed the reaction, and the month of resolution was defined as the month in which the patient first noticed that the symptoms had resolved. Assessment of ISRs was based on patient self‐reports rather than direct physician examination. In this study, ISRs were defined as symptoms occurring around the injection site after SC administration, including erythema, swelling, and itching. In contrast, reactions considered to be related to mechanical stimulation from injection, such as pain, induration, or SC bleeding, were excluded from the definition of ISRs. Because this was a retrospective study, the severity of ISRs was not evaluated using a standardized quantitative grading system. Instead, the clinical relevance of ISRs was assessed based on their clinical course and categorized as cases managed with observation alone, cases requiring symptomatic treatment, and cases in which ISRs led to discontinuation of SC vedolizumab. Pharmacological symptomatic treatment for ISRs was provided at the discretion of the attending physician. At the time of switching to SC vedolizumab, all patients received two sessions of injection training by IBD nurses. After the occurrence of ISRs, additional instructions were provided on a case‐by‐case basis, including rotation of injection sites and allowing the drug to reach room temperature before administration.

### 2.3 Statistical Analysis

Data were presented as number (%) or median (interquartile range [IQR]). The Wilcoxon signed‐rank test was used to examine continuous variables. The McNemar's test or the exact McNemar's test was utilized to assess categorical variables at baseline and 12 months after switching to SC vedolizumab. At baseline, pMayo score, CRP, and FCP were available for all patients. At 12 months, pMayo score and CRP were missing in two patients (one due to treatment discontinuation and one censored due to death). FCP values at 12 months were missing in three patients: one due to treatment discontinuation, one censored due to death, and one due to failure to submit a stool sample. Analyses were conducted using available‐case data without imputation [20]. Kaplan–Meier survival curves were used to estimate drug persistence rates. Treatment discontinuation for any reason was defined as the event. The time to the event was defined as the time from switching to SC vedolizumab to treatment discontinuation. Patients were censored at the last known date of clinical follow‐up if treatment discontinuation had not occurred, including cases in which patients were transferred to another hospital or were lost to follow‐up before 12 months after switching. One patient died during the observation period and was treated as a censored case because the death was not considered related to SC vedolizumab treatment. *p* < 0.05 indicated statistically significant differences. Stata version 18.0 (StataCorp LLC, College Station, TX, USA) was used for all statistical analyses.

### 2.4 Ethical Considerations

This study was approved by the Institutional Ethics Committee (approval no. 2021CS‐1), and it was performed based on the principles of the Declaration of Helsinki. The need for written informed consent was waived due to the retrospective nature of the study and the use of anonymized data.

## 3 Results

### 3.1 Characteristics of the Patients

This study included 48 patients who switched from IV to SC vedolizumab between June 2024 and November 2024. Table 1 shows the baseline demographic and clinical characteristics of the participants. The median duration of previous vedolizumab use was 36.5 (IQR: 22.8–44.9) months. Regarding clinical disease activity, the median pMayo score was 0 (IQR: 0–0). For biochemical disease activity, the median CRP level was 0.03 (IQR: 0.01–0.07) mg/dL, and the median FCP level was 49.7 (IQR: 18.5–96.5) μg/g. At baseline, the concomitant medications included 5‐aminosalicylates in 33 patients (69%) and corticosteroids in one (2%), which were prescribed for bullous pemphigoid rather than UC.

**TABLE 1:** Baseline demographic and clinical characteristics of patients switching from IV to SC vedolizumab.

Characteristics	All patients (*N* = 48)
Sex	
Male	26 (54)
Female	22 (46)
Age (years)	49 (32–55.5)
Disease duration (years)	7.9 (4.5–13.9)
Disease extent	
Extensive colitis or pancolitis	34 (71)
Left‐sided colitis	12 (25)
Proctitis	2 (4)
Partial Mayo score	0 (0–0)
Biochemical parameters	
CRP level (mg/dL)	0.03 (0.01–0.07)
FCP level (μg/g)	49.7 (18.5–96.5)
Duration of previous vedolizumab use (months)	36.5 (22.8–44.9)
Concomitant medications	
5‐aminosalicylates	33 (69)
Corticosteroid	1 (2)
Budesonide	0 (0)
Immunomodulator	0 (0)
Calcineurin inhibitor	0 (0)
History of treatment with advanced therapy	
Advanced therapy naïve	44 (92)
Advanced therapy experienced	4 (8)
Number of advanced therapy	
0	44 (92)
1	3 (6)
2	1 (2)
Previous history of advanced therapies	
Infliximab	4 (8)
Adalimumab	1 (2)
Baseline clinical and biochemical remission rates	
Clinical remission^a^	45 (94)
CRP levels < 0.3 mg/dL	45 (94)
FCP levels < 150 μg/g	42 (88)

*Note:* Values were presented as number (%) or median (interquartile range).

Abbreviations: CRP, C‐reactive protein; FCP, fecal calprotectin; IV, intravenous; SC, subcutaneous.

^a^ Clinical remission was defined as a pMayo score of ≤ 2 with a rectal bleeding subscore of 0.

### 3.2 Changes in Biomarker Levels and Clinical Activity at 12 Months After the Switch

**FIGURE 1:**
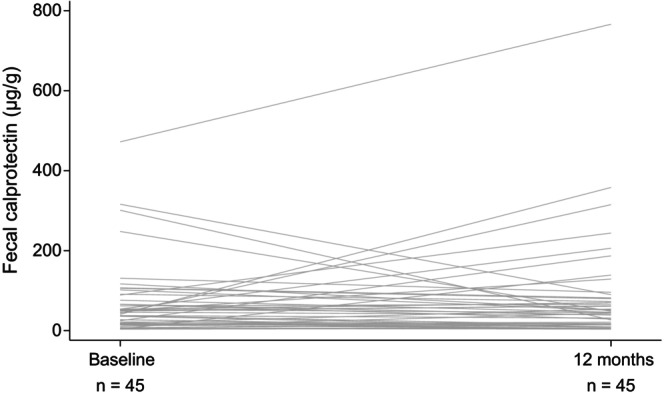
Changes in FCP levels from baseline to 12 months after switching from IV to SC vedolizumab. Individual patient trajectories are shown using paired data from 45 patients with available measurements at both time points. The median FCP levels did not significantly change between baseline and 12 months (46.0 vs. 46.0 μg/g, *p* = 0.694). FCP, fecal calprotectin; IV, intravenous; SC, subcutaneous.

**FIGURE 2:**
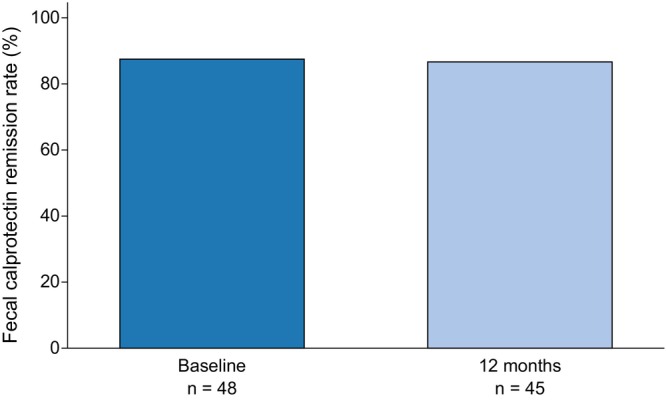
Changes in FCP remission rates from baseline to 12 months after switching from IV to SC vedolizumab. The proportion of patients achieving FCP remission was 42/48 (88%) at baseline and 39/45 (87%) at 12 months. Paired comparisons were performed in 45 patients with available FCP measurements at both time points (41/45, 91% vs. 39/45, 87%, *p* = 0.727). FCP, fecal calprotectin; IV, intravenous; SC, subcutaneous.

Paired analyses were performed in 45 patients for FCP and in 46 patients for CRP and pMayo score. There were no significant differences in terms of median FCP levels between baseline and 12 months after switching to SC vedolizumab (46.0 vs. 46.0 μg/g, *p* = 0.694) (Figure 1). Similarly, the median CRP levels showed no significant change between baseline and 12 months (0.03 vs. 0.04 mg/dL, *p* = 0.553). The biomarker remission rates also did not change. In particular, the FCP remission rates were 41/45 (91%) at baseline and 39/45 (87%) at 12 months (*p* = 0.727), and the CRP remission rates were 43/46 (93%) and 43/46 (93%) (*p* = 1.000), respectively (Figure 2).

Clinical disease activity, assessed using the median pMayo score, remained stable (0 vs. 0, *p* = 0.500), and the clinical remission rates similarly showed no significant difference between baseline and 12 months (43/46, 93% vs. 42/46, 91%, *p* = 1.000).

### 3.3 Drug Persistence at 12 Months After the Switch

**FIGURE 3:**
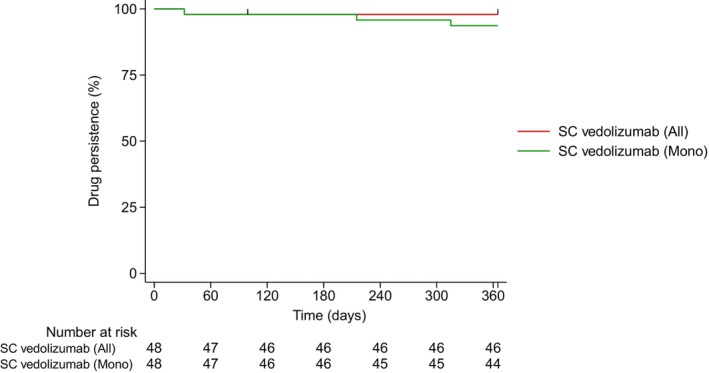
Kaplan–Meier curves for drug persistence after switching from IV to SC vedolizumab. SC vedolizumab (All) represents overall persistence. Meanwhile, SC vedolizumab (Mono) indicates persistence without additional treatment. At 12 months, the overall persistence rate was 98%, and the persistence rate without additional treatment was 94%. During follow‐up, treatment discontinuation occurred in one patient due to disease worsening. One patient died from another disease and was treated as a censored case. IV, intravenous; SC, subcutaneous.

At 12 months after the switch, the persistence rate of SC vedolizumab was 98%, and the persistence rate without additional treatment was 94% (Figure 3). During follow‐up, treatment discontinuation occurred in one patient due to disease worsening. One patient died from another disease during follow‐up and was treated as a censored case. Two additional patients continued to receive SC vedolizumab but required concomitant MMX‐budesonide. Notably, none of the patients discontinued SC vedolizumab due to switching back to the IV formulation.

### 3.4 Adverse Events

Table 2 shows all the observed adverse events. In addition to ISRs, adverse events such as headache and fatigue were observed in 16 patients (33%). Although a causal association with SC vedolizumab could not be definitively excluded, none of these events were considered likely or probably related to SC vedolizumab based on clinical judgment. SC vedolizumab was generally well tolerated, with ISRs being the primary treatment‐related concern.

**TABLE 2:** All observed adverse events during SC vedolizumab treatment.

Adverse events	All patients (*N* = 48)
ISRs	29 (60)
Erythema	20 (42)
Swelling	13 (27)
Itching	20 (42)
Common cold	5 (10)
Ureteral stone	2 (4)
Fatigue	2 (4)
Death	1 (2)
Malignancy^a^	1 (2)
Acquired hemophilia	1 (2)
Tuberculosis	1 (2)
Transient ischemic attack	1 (2)
Appendicitis	1 (2)
External otitis	1 (2)
Headache	1 (2)

*Note:* Values were expressed as number (%).

Abbreviation: ISRs, injection‐site reactions.

^a^ Malignancy was glottic carcinoma.

### 3.5 ISRs

**FIGURE 4:**
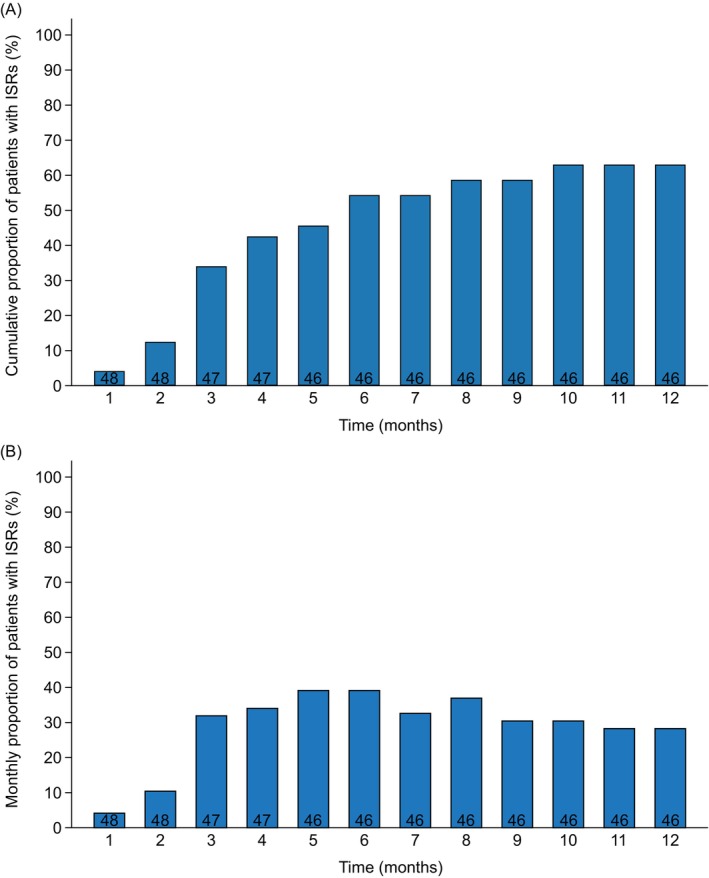
Time course and persistence of ISRs after switching from IV to SC vedolizumab. Monthly denominators (number of patients receiving SC vedolizumab during each month of follow‐up) are shown within the bars. Patients were excluded from the denominator after treatment discontinuation or death. (A) Cumulative proportion of patients with ISRs after switching from IV to SC vedolizumab. Most patients developed ISRs between 3 and 6 months after switching. (B) Monthly proportion of patients with ISRs after switching from IV to SC vedolizumab. The percentages represent the period prevalence, defined as the proportion of patients who experienced at least one ISR during each month, including transient events. ISR onset and resolution months were assigned based on patient self‐reports, corresponding to the months when the reaction was first noticed and when the symptoms were first noticed to have resolved. The percentage peaked at 6 months, and the ISRs typically improved or resolved by the end of the 12‐month follow‐up. ISRs, injection‐site reactions; IV, intravenous; SC, subcutaneous.

In total, 29 patients (60%) exhibited ISRs, including erythema (*n* = 20), swelling (*n* = 13), and itching (*n* = 20), at any time during follow‐up. Figure 4A shows the cumulative proportion of patients with ISRs. More than 60% of these reactions developed between 3 and 6 months after switching. The median duration was 4 (IQR: 2–8) months, and the proportion of affected patients peaked at 6 months (Figure 4B). More than half of the ISRs improved or completely resolved by 12 months. Consequently, only 11 patients (23%) experienced persistent ISRs at that time (erythema: *n* = 10, swelling: *n* = 6, and itching: *n* = 9). Importantly, most ISRs were managed with observation alone, and only one patient required topical antihistamine treatment. No patients discontinued SC vedolizumab because of ISRs.

## 4 Discussion

This study evaluated the efficacy and safety of switching from IV to SC vedolizumab in patients with UC in Japan. Both clinical and biomarker disease activity remained stable after switching, with no significant changes from baseline. ISRs occurred after switching from IV to SC vedolizumab, affecting approximately 60% of patients. However, more than half showed improvement or complete resolution by 12 months.

In our study, disease activity based on both clinical and biomarker assessments remained stable after switching, and the corresponding remission rates were also maintained. Previous studies conducted in Western countries have reported higher vedolizumab trough levels with the SC formulation than with the IV formulation. Nonetheless, there were no significant changes in disease activity before and after switching [12, 13]. Our cohort included a large proportion of patients who were already in remission at the time of switching. Both clinical and biomarker remission rates were high—with an overall rate of approximately 90%—which was higher than those reported in previous studies. Nevertheless, there were no significant changes before and after switching, and our findings are consistent with those of previous reports. Our findings suggest that disease activity was maintained after switching from IV to SC vedolizumab in Asian patients with UC.

Remarkably, our study revealed a high persistence rate (98%) at 12 months. Previous studies have reported persistence rates of 81.1%–88.5% at 12 months and 73.6% at 18 months [12, 14, 15]. In Korea, the persistence rate at 6 months was 71.3%, which was also lower than that observed in our cohort [16]. The higher persistence rate in our study might be attributed to the high proportion of patients in remission at the time of switching, as previous reports have shown that nonremission at switching is associated with discontinuation of SC vedolizumab [15]. These findings suggest that switching from IV to SC vedolizumab was generally well tolerated and associated with high treatment persistence in appropriately selected patients, particularly those who are in remission at the time of switching.

In our study, none of the patients developed serious adverse events related to switching from IV to SC vedolizumab. Previous studies have shown that 24.4% of patients with UC presented with adverse events excluding ISRs [12]. In the current study, adverse events other than ISRs were observed in 33% of the participants, which was slightly higher but generally consistent with previous reports. Moreover, no adverse events other than ISRs were clearly attributed to the SC formulation. However, the incidence of ISRs in our cohort was higher than that reported in previous studies (60% vs. 11%–42%) [11, 12, 13]. This difference may be partly explained by our detailed review of routine clinical records, including nurses' interviews and physicians' chart notes, which enabled the detection of even mild and transient ISRs that might have been underreported elsewhere. In addition, our study had a relatively long observation period (12 months) and a high treatment persistence rate, which might have increased the incidence of ISRs. These factors could have contributed to the higher ISR rate observed and should be further investigated in future research.

In clinical practice, switching back from SC to IV vedolizumab is occasionally attempted in patients who develop ISRs. However, such switching procedures may occasionally induce the so‐called “tricky reactions,” as described in a previous report, including urticaria, lip swelling, hypotension, and erythema at previous SC injection sites [21]. Importantly, in our study, more than half of the ISRs improved or completely resolved spontaneously within 12 months without switching back to IV vedolizumab. To the best of our knowledge, this is the first real‐world study showing the clinical course of ISRs along with the switching from IV to SC vedolizumab. These findings provide novel insights into switching‐back strategies, indicating that most ISRs are self‐limiting and that switching back to IV formulation solely because of ISRs should be cautiously considered.

The current study has several limitations. Its retrospective design might have introduced information bias, as data collection relied on medical records and all mild adverse events might not have been identified. Moreover, a small amount of missing data for key outcomes (including FCP at 12 months) was present due to treatment discontinuation, censoring, or lack of measurement, which may have introduced some degree of bias. Furthermore, death was treated as a censoring event in the persistence analysis because it was not considered related to SC vedolizumab; however, persistence estimates may vary depending on whether death is treated as censoring or as a treatment discontinuation event. In addition, this study was conducted at a single center in Japan with a relatively small sample size, which could have limited the generalizability of the findings to other populations or healthcare settings. Another limitation is the absence of vedolizumab trough level and antidrug antibody measurements, which prevented a detailed pharmacokinetic or immunogenic assessment. Further, the observation period was limited to 12 months; thus, long‐term persistence or late‐onset ISRs might not have been fully detected. These limitations should be considered when interpreting the results. Nevertheless, despite these limitations, our findings regarding the clinical course of ISRs after switching from IV to SC vedolizumab in clinical settings are unique and can provide novel insights into switching back strategies that are applicable across different regions.

In conclusion, clinical and biomarker disease activity was maintained after switching from IV to SC vedolizumab in Asian patients with UC. ISRs may develop after switching from IV to SC; however, most of them are self‐limiting. Thus, switching back to IV formulation solely because of ISRs should be cautiously considered to prevent the potential “tricky reactions.”

## Funding

The authors have nothing to report.

## Ethics Statement

The study conformed to the principles of the Declaration of Helsinki and current ethical regulations, with approval from the institutional ethics committee (No. 2021CS‐1).

## Consent

Written informed consent was waived because the data were anonymized and retrospectively analyzed.

## Conflicts of Interest

The authors declare no conflicts of interest.

## Data Availability

The data that support the findings of this study are available on request from the corresponding author. The data are not publicly available due to privacy or ethical restrictions.

## References

[1] D. K. Podolsky , “Inflammatory Bowel Disease,” New England Journal of Medicine 347 (2002): 417–429. 12167685 10.1056/NEJMra020831

[2] J. Cosnes , C. Gower‐Rousseau , P. Seksik , and A. Cortot , “Epidemiology and Natural History of Inflammatory Bowel Diseases,” Gastroenterology 140 (2011): 1785–1794. 21530745 10.1053/j.gastro.2011.01.055

[3] N. A. Molodecky , I. S. Soon , D. M. Rabi , et al., “Increasing Incidence and Prevalence of the Inflammatory Bowel Diseases With Time, Based on Systematic Review,” Gastroenterology 142 (2012): 46–54. 22001864 10.1053/j.gastro.2011.10.001

[4] T. Raine , S. Bonovas , J. Burisch , et al., “ECCO Guidelines on Therapeutics in Ulcerative Colitis: Medical Treatment,” Journal of Crohn's & Colitis 16 (2022): 2–17. 10.1093/ecco-jcc/jjab178 34635919

[5] B. G. Feagan , P. Rutgeerts , B. E. Sands , et al., “Vedolizumab as Induction and Maintenance Therapy for Ulcerative Colitis,” New England Journal of Medicine 369 (2013): 699–710. 23964932 10.1056/NEJMoa1215734

[6] E. V. Loftus, Jr. , B. G. Feagan , R. Panaccione , et al., “Long‐Term Safety of Vedolizumab for Inflammatory Bowel Disease,” Alimentary Pharmacology & Therapeutics 52 (2020): 1353–1365. 32876349 10.1111/apt.16060 PMC7540482

[7] K. L. Stoner , H. Harder , L. J. Fallowfield , and V. A. Jenkins , “Intravenous Versus Subcutaneous Drug Administration: Which Do Patients Prefer? A Systematic Review,” Patient 8 (2015): 145–153. 10.1007/s40271-014-0075-y 25015302

[8] P. M. Overton , N. Shalet , F. Somers , and J. A. Allen , “Patient Preferences for Subcutaneous Versus Intravenous Administration of Treatment for Chronic Immune System Disorders: A Systematic Review,” Patient Preference and Adherence 15 (2021): 811–834. 33907384 10.2147/PPA.S303279 PMC8064718

[9] G. Burdge , A. Hardman , I. Carbery , G. Broglio , D. Greer , and C. P. Selinger , “Uptake of a Switching Program for Patients Receiving Intravenous Infliximab and Vedolizumab to Subcutaneous Preparations,” Journal of Clinical Medicine 11 (2022): 5669. 36233537 10.3390/jcm11195669 PMC9571673

[10] W. J. Sandborn , F. Baert , S. Danese , et al., “Efficacy and Safety of Vedolizumab Subcutaneous Formulation in a Randomized Trial of Patients With Ulcerative Colitis,” Gastroenterology 158 (2020): 562–572. 31470005 10.1053/j.gastro.2019.08.027

[11] E. Ventress , D. Young , S. Rahmany , et al., “Transitioning From Intravenous to Subcutaneous Vedolizumab in Patients With Inflammatory Bowel Disease [TRAVELESS],” Journal of Crohn's & Colitis 16 (2022): 911–921. 10.1093/ecco-jcc/jjab224 PMC9383144 34935945

[12] V. Bergqvist , J. Holmgren , D. Klintman , and J. Marsal , “Real‐World Data on Switching From Intravenous to Subcutaneous Vedolizumab Treatment in Patients With Inflammatory Bowel Disease,” Alimentary Pharmacology & Therapeutics 55 (2022): 1389–1401. 35470449 10.1111/apt.16927 PMC9322578

[13] A. Volkers , T. Straatmijer , M. Duijvestein , et al., “Real‐World Experience of Switching From Intravenous to Subcutaneous Vedolizumab Maintenance Treatment for Inflammatory Bowel Diseases,” Alimentary Pharmacology & Therapeutics 56 (2022): 1044–1054. 35869807 10.1111/apt.17153 PMC9540102

[14] S. H. Lim , B. Gros , E. Sharma , et al., “Safety, Effectiveness, and Treatment Persistence of Subcutaneous Vedolizumab in IBD: A Multicenter Study From the United Kingdom,” Inflammatory Bowel Diseases 30 (2024): 1284–1294. 37603730 10.1093/ibd/izad166

[15] T. H. Wiken , M. L. Høivik , K. Anisdahl , et al., “Subcutaneous Vedolizumab Treatment in a Real‐World Inflammatory Bowel Disease Cohort Switched From Intravenous Vedolizumab: Eighteen‐Month Prospective Follow‐Up Study,” Crohn's & Colitis 360 6 (2024): otae013. 10.1093/crocol/otae013 PMC10972549 38544907

[16] Y. K. Jun , Y. Choi , C. M. Shin , et al., “Real‐World Experience of Switching From Intravenous to Subcutaneous Vedolizumab in Korean Patients With Inflammatory Bowel Disease,” Gut and Liver 20 (2025): 266–274, 10.5009/gnl250188. 40958654 PMC12989668

[17] F. Magro , P. Gionchetti , R. Eliakim , et al., “Third European Evidence‐Based Consensus on Diagnosis and Management of Ulcerative Colitis. Part 1: Definitions, Diagnosis, Extra‐Intestinal Manifestations, Pregnancy, Cancer Surveillance, Surgery, and Ileo‐Anal Pouch Disorders,” Journal of Crohn's & Colitis 11 (2017): 649–670. 10.1093/ecco-jcc/jjx008 28158501

[18] K. W. Schroeder , W. J. Tremaine , and D. M. Ilstrup , “Coated Oral 5‐Aminosalicylic Acid Therapy for Mildly to Moderately Active Ulcerative Colitis. A Randomized Study,” New England Journal of Medicine 317 (1987): 1625–1629. 3317057 10.1056/NEJM198712243172603

[19] S. Singh , A. N. Ananthakrishnan , N. H. Nguyen , et al., “AGA Clinical Practice Guideline on the Role of Biomarkers for the Management of Ulcerative Colitis,” Gastroenterology 164 (2023): 344–372. 36822736 10.1053/j.gastro.2022.12.007

[20] L. Thabane , L. Mbuagbaw , S. Zhang , et al., “A Tutorial on Sensitivity Analyses in Clinical Trials: The What, Why, When and How,” BMC Medical Research Methodology 13 (2013): 92. 23855337 10.1186/1471-2288-13-92 PMC3720188

[21] N. Richard , L. Vuitton , and M. Fumery , “Letter: Tricky Reactions to Switch Back From Subcutaneous to Intravenous Vedolizumab in Patients With Inflammatory Bowel Disease,” Alimentary Pharmacology & Therapeutics 57 (2023): 741–742. 36821749 10.1111/apt.17395

